# Comparative efficacy and safety of nitinol vs. novel fully biodegradable occluders for transcatheter patent foramen ovale closure in migraine treatment: a retrospective cohort study

**DOI:** 10.3389/fmed.2025.1613687

**Published:** 2025-07-02

**Authors:** Rui-lin Li, Jing-juan Huang, Jie Ming, Ying Hao, Wei Wen, Yun-li Shen, Li Lin, Lin-xiang Lu

**Affiliations:** ^1^Department of Cardiology, Shanghai East Hospital, Tongji University School of Medicine, Shanghai, China; ^2^Department of Cardiology, Shanghai Chest Hospital, Shanghai Jiao Tong University, Shanghai, China; ^3^Department of Clinical Laboratory, Shanghai East Hospital, Tongji University School of Medicine, Shanghai, China

**Keywords:** patent foramen ovale, biodegradable occluder, nitinol occluder, migraine, MIDAS score

## Abstract

**Background:**

Transcatheter closure of patent foramen ovale (PFO) has shown potential therapeutic benefits for clinical symptoms in selected patients with migraine. Nevertheless, the comparative effectiveness and safety of traditional nitinol vs. fully biodegradable occluders remain to be fully elucidated and warrant further investigation.

**Materials and methods:**

This retrospective cohort study included 158 migraine patients with a confirmed patent foramen ovale (PFO) and a grade II–III right-to-left shunt (RLS) as determined by contrast-enhanced transthoracic echocardiography (cTTE), who underwent transcatheter PFO closure at Shanghai East Hospital, Tongji University, between October 2023 and January 2024. Based on the occluder type, patients were categorized into a nitinol group (*n* = 77) or a biodegradable group (*n* = 81). Clinical baseline characteristics, echocardiographic parameters, procedural data, safety outcomes, residual right-to-left shunt (RLS) grades, and migraine severity assessed using the Migraine Disability Assessment Scale (MIDAS) were compared between groups. The primary outcome was migraine relief (≥50% reduction in MIDAS score) following the procedure. Secondary outcomes included the incidence of safety-related events and the rate of residual right-to-left shunt.

**Results:**

Both the nitinol group and biodegradable groups showed significant reductions in MIDAS scores post-procedure with no significant difference in migraine relief between groups (*P* = 0.644, Kaplan-Meier analysis). Both occluders showed a high procedural success rate (100%) and similar safety profiles, with low rates of perioperative complications. The biodegradable occluder exhibited progressive degradation, with a significant reduction in size by 12 months, while maintaining closure efficacy. Residual right-to-left shunting was minimal in both groups, with RLS grades 0 and 1 observed in 82.35% of patients in the nitinol group and 78.87% in the bioabsorbable group at the 12-month follow-up.

**Conclusion:**

Both biodegradable and nitinol PFO occluders were equally effective in alleviating migraine symptoms, with excellent procedural success and safety profiles. The biodegradable occluder demonstrated effective closure and gradual degradation, offering a promising alternative to nitinol occluders, especially for patients concerned about long-term foreign body implantation. These findings support the clinical utility of both occluder types in PFO-related migraine treatment, with individualized device selection based on patient preferences and clinical factors.

## 1 Introduction

Migraine is a prevalent chronic neurological disorder affecting ~15% of the global population, with a notably higher prevalence in women (1). It is characterized by recurrent, debilitating headaches often accompanied by nausea, photophobia, and phonophobia, significantly affecting the quality of life and presenting a considerable public health challenge (2). In recent years, numerous investigations have identified a significant link between patent foramen ovale (PFO) and migraine (3–6), prompting efforts to explore the therapeutic potential of transcatheter PFO closure in migraine management.

PFO is the most common congenital cardiac anomaly in adults, persisting in ~20%−35% of the population, it results from the incomplete closure of the fetal foramen ovale, creating a potential right-to-left atrial shunt (7, 8). This shunting allows paradoxical emboli and vasoactive substances, such as serotonin and bradykinin, to bypass the pulmonary circulation and enter the systemic circulation unfiltered (9, 10). Proposed mechanisms linking PFO to migraine include cortical spreading depression triggered by microemboli, unfiltered vasoactive substances inducing vascular dysregulation, impaired cerebral autoregulation, and genetic predisposition (11–14).

The therapeutic benefit of PFO closure in migraine management has been explored in numerous observational studies and clinical trials. Since the initial report of Swiss divers experiencing migraine relief following patent foramen ovale (PFO) closure (15), the association between PFO and migraine has attracted both interest and skepticism. To investigate the potential benefits of PFO closure for migraine symptoms, several small randomized trials have been conducted, including the MIST trial (Migraine Intervention with STARFlex Technology) (16), the ESCAPE Migraine trial (17), and more recently, the PRIMA trial (Percutaneous Closure of PFO in Migraine with Aura Patients) (18), as well as the PREMIUM trial (Prospective Randomized Investigation to Evaluate Incidence of Headache Reduction in Migraine Patients With PFO Using the Amplatzer PFO Occluder vs. Medical Management) (19). As reported in the PREMIUM trial, PFO closure did not achieve the primary endpoint of reducing the responder rate in patients with frequent migraine. Nevertheless, patients in the closure group exhibited a significantly greater reduction in headache days, indicating a consistent trend toward symptom improvement. However, heterogeneity in outcomes has highlighted the need for further research to identify specific patient subgroups that may benefit most from this intervention (20).

While nitinol occluders are effective, they may trigger migraines via microthrombi or nickel hypersensitivity (21). In recent decades, the advancement of biodegradable occluder technology has emerged as a promising approach for transcatheter PFO closure, owing to its enhanced biocompatibility and ability to gradually degrade within the body (22–25). However, the relative efficacy and safety profiles of conventional nitinol occluders compared to novel fully biodegradable devices have not been fully established and require further comprehensive evaluation.

This retrospective cohort study aims to address this gap by evaluating the impact of these two device types on migraine outcomes during a 1-year follow-up period. By using the Migraine Disability Assessment Questionnaire (MIDAS), a validated assessment tool, this study aims to comprehensively compare the effectiveness of these devices in reducing the severity of migraines. Simultaneously evaluate the safety and effectiveness of degradable PFO occluders. These findings will contribute to the growing understanding of PFO closure as a targeted intervention for migraine management and inform clinical decision-making for device selection in patients with PFO-associated migraines.

## 2 Materials and methods

### 2.1 Study design and population

This retrospective cohort study included migraine patients diagnosed with PFO who were admitted to Shanghai East Hospital, Tongji University, between October 2023 and January 2024. Inclusion Criteria: (1) patients aged 18–75 years; (2) preoperative screening for PFO was conducted using contrast-enhanced transthoracic echocardiography (cTTE), demonstrating a right-to-left shunt (RLS) of grade II–III. The diagnosis was subsequently confirmed by either transesophageal echocardiography (TEE) or intracardiac echocardiography (ICE); (3) migraine diagnosis established according to the third edition of the International Classification of Headache Disorders; (4) failure to benefit from conventional drug therapy for migraines; (5) consent for percutaneous PFO closure; (6) thorough preoperative evaluation by a multidisciplinary team, including cardiologists, neurologists, and imaging specialists. Exclusion Criteria: (1) other known causes of migraine or cerebral infarction of identifiable origin; (2) contraindications to antiplatelet or anticoagulation therapy; (3) large cerebral infarction within the past 4 weeks; (4) systemic or local infections, sepsis, or intracardiac thrombosis; (5) planned pregnancies, current pregnancy, or breastfeeding; (6) pulmonary arterial hypertension or PFO serving as a survival pathway. (7) severe comorbidities or systemic diseases that could affect study outcomes (e.g., severe cardiovascular disease, renal failure, autoimmune disorders, abnormal liver function, thyroid disorders, or diabetes mellitus); (8) participation in other clinical trials that could interfere with this study.

The Ethics Committee of Shanghai East Hospital granted approval for the study (approval number: 2024YS-181). The research has been recorded in the Chinese Clinical Trial Registry (registration number: ChiCTR2500097046). All procedures adhered to the Declaration of Helsinki and its subsequent amendments. Written informed consent was obtained from all participants or their legal guardians prior to inclusion in the study.

### 2.2 Intervention

All patients who underwent occlusion procedures met the recommended indications for percutaneous PFO interventional occlusion therapy (26), Two types of nitinol occluders are used: the AMPLATZER™ occlusion occluder (Abbott; occluder model and size: 18/18 mm; 18/25 mm), the oxidized membrane single-rivet occluder (Shanghai Shape Memory Alloy Materials Co., Ltd.; occluder model and size: 18/18 mm; 24/24 mm; 28/28 mm; 34/34 mm), and the fully biodegradable PFO occluder (Memosorb^®^ PFO occluder, Shanghai Shape Memory Alloy Materials Co., Ltd.; occluder model and size: 18/18 mm; 24/24 mm; 28/28 mm; 34/34 mm). Biodegradable occluders were composed of fully resorbable materials, while nitinol occluders consisted of nitinol-based structures. The Memosorb^®^ PFO occluder (Shanghai Shape Memory Alloy Co., Ltd, Shanghai, China) is a fully biodegradable device designed for the percutaneous closure of PFO. The occluder is made of a polylactic acid (PLA)-based framework and consists of a double-disk structure. The frame is constructed from polydioxanone (PDO) monofilament, and both disks are filled with poly-L-lactic acid (PLLA) membranes. This unique design enables the device to be delivered in a tube-like configuration, which can be transformed into an umbrella-like shape for defect closure. The selection of PFO occluder model and size is primarily guided by the anatomical characteristics of the PFO, clinical complexity, individual patient factors, and patient preferences to ensure optimal closure outcomes and satisfaction. PFO closure was performed under fluoroscopic guidance, supplemented by transesophageal echocardiography (TEE) or intracardiac echocardiography (ICE) for real-time imaging and procedural monitoring.

The Memosorb^®^ occluder features an advanced delivery system, which consists of an external pushing tube and an internal wire rope. This system allows the device to be transformed from the delivery tube shape to the fully expanded umbrella shape for optimal PFO closure. A locking system securely holds the device in place during deployment, ensuring it remains in the correct position. The device's pentagonal skeleton connects the two disks, eliminating the traditional “waist” and making it suitable for complex PFOs, such as those with narrow paths or multi-fenestrated defects.

The procedure begins with femoral vein cannulation (or another suitable access point) to gain vascular access. A long sheath is inserted into the femoral vein, which will be used to deliver the occluder into the heart. Fluoroscopy and ultrasound guidance are used to ensure accurate placement. In clinical practice, the selection of occluder size for PFO closure is not determined solely by the measured PFO diameter. Instead, it must also take into account complex anatomical features that may impact closure efficacy. These complex PFOs include long-tunnel PFOs (tunnel length ≥8 mm), the presence of an atrial septal aneurysm (ASA), septum secundum thickness ≥10 mm, prominent Eustachian valve or Chiari network, multiple left atrial exit sites, or anatomical distortion caused by a dilated aortic root (27). Careful evaluation of these characteristics is essential to ensure adequate occluder coverage and to achieve effective closure. The occluder is introduced into the heart via the loading sheath and delivery sheath; upon its release from the delivery sheath, the left disk of the occluder initially assumes a spherical shape (Supplementary Videos). It is subsequently molded into a disk by retracting the shape line through the shape line. After confirming the shape of the left disk, the right disk is subsequently deployed. The entire occluder adopts a “double-umbrella” configuration as the shape line is retracted while concurrently advancing the delivery cable. When the knot of the shape line is extracted from the connecting rivet of the occluder, it secures the device in its final “double-umbrella” form, anchoring it firmly. Subsequently, the shape line was detached from the occluder. A series of videos of the release of the occluder were shown in Supplementary material.

According to the selected occluder, patients were divided into nitinol group and bioabsorbable group. Procedural success and complications were recorded. Routine transthoracic echocardiography (TTE) and 12-lead electrocardiography were performed after the procedure. Both groups received the same antiplatelet regimen. No loading dose of antiplatelet therapy was administered either before or after the PFO closure procedure. Starting from the first postoperative day, all patients received dual antiplatelet therapy (DAPT) consisting of aspirin 100 mg once daily and clopidogrel 75 mg once daily. This regimen was maintained for 6 months post-procedure. After this initial period, patients were switched to monotherapy with either aspirin 100 mg once daily or clopidogrel 75 mg once daily for an additional 6 months, resulting in a total antiplatelet therapy duration of 12 months. Antiplatelet therapy was discontinued after 12 months if no thrombotic events were observed during follow-up.

### 2.3 Follow-up and outcome measures

This comprehensive follow-up schedule enabled detailed evaluation of baseline characteristics, postoperative efficacy, safety, and short-term device performance.

Baseline patient information, including gender, age, stature, body mass, blood pressure, and pulse rate, blood test results, the echocardiographic parameters including baseline cardiac chamber dimensions, preserved left ventricular function, and comparable pulmonary pressures were collected prior to the procedure. The morphology and size of the PFO were assessed preoperatively using transesophageal echocardiography (TEE) and intraoperatively measured with intracardiac echocardiography (ICE). Follow-up data were obtained at 1, 3, 6, and 12 months post-procedure. Residual right-to-left shunt (RLS) grades were assessed at 6 and 12 months follow-up visit using contrast transthoracic echocardiography (cTTE). The severity of migraines was evaluated before the procedure and at 3, 6, and 12 months follow-up visit post-procedure using the Migraine Disability Assessment Questionnaire (MIDAS). The areas of the occluder disks were measured in four-chamber views using transthoracic echocardiography (TTE) after discharge and at 1, 3, 6, and 12 months post-procedure. The primary endpoint was to compare migraine relief (≥50% reduction in MIDAS score) between the nitinol group and the the Migraine Disability Assessment (MIDAS) bioabsorbable group following the procedure. In clinical practice, both a ≥50% reduction in monthly headache days (MHD) and a ≥30% reduction in score are commonly used to evaluate patient improvement. However, given that PFO closure is an invasive, device-based intervention, we believe that a more stringent efficacy threshold is warranted when assessing its therapeutic impact. Considering that the MIDAS score includes the specific number of days on which migraine affects a patient's daily life and work. Therefore, we opted to define significant improvement as a ≥50% reduction in MIDAS score, aligning the evaluation with the invasive nature and long-term intent of PFO closure (28). The secondary objectives was to evaluate the short-term safety following the implantation of the fully biodegradable PFO occluder by assessing Serious Adverse Device Events (SADEs) and the rate of residual right-to-left shunt. SADEs included sudden cardiac arrest, cardiac tamponade, endocarditis, thrombus formation on the device, occluder detachment, vascular complications and new arrhythmias, or any cardiac or general event that could potentially be attributed to the implanted device or the procedure.

### 2.4 Statistical analysis

Normally distributed continuous variables are expressed as mean ± standard deviation, non-normally distributed continuous variables are expressed as median (interquartile range, IQR), and count data are expressed as frequencies and/or percentages. Comparisons of continuous variables for normality were made using the two independent samples Student's *t*-test, and comparisons of continuous variables for non-normality were made using the Mann-Whitney *U* test. Data comparisons for categorical variables were made using Pearson's Chi-square test, corrected Chi-square test, or Fisher's exact probability method. Survival analysis was performed using Kaplan-Meier curves, with survival defined as the time between the first postoperative day of follow-up and the event. Patients who were lost to follow-up or died of unrelated causes were excluded. Differences in survival curves were compared using the Log-rank test or the Breslow test.

Statistical evaluations were executed utilizing SPSS 27.0 software (version 22.0; IBM Corporation, Armonk, NY, USA), while certain findings were illustrated with Graphpad Prism 10.4; all significance assessments were carried out as two-tailed, with a *P*-value threshold of under 0.05 considered statistically significant.

## 3 Results

### 3.1 Baseline characteristics and procedure

**Figure 1:**
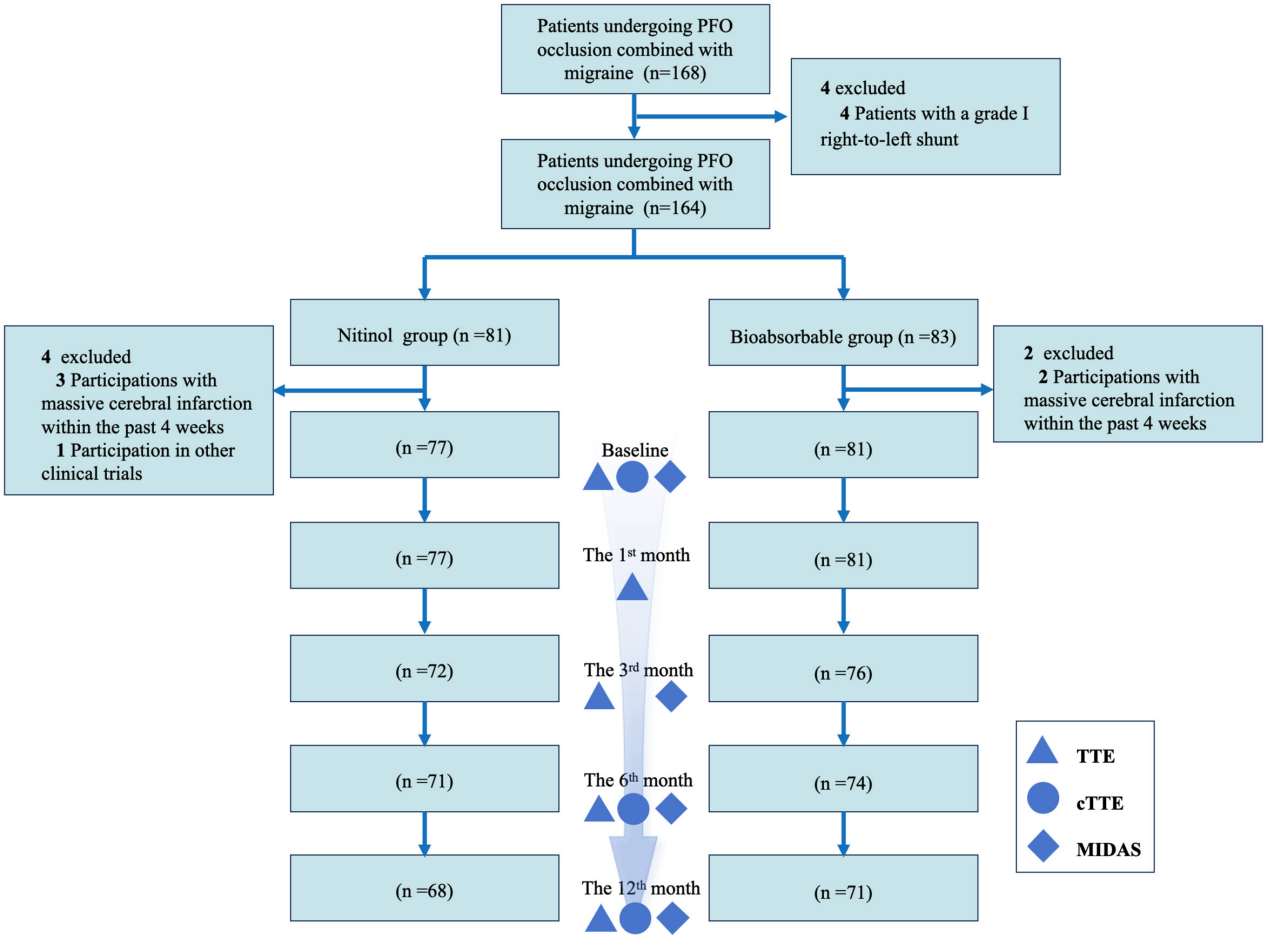
Study flowchart. PFO, patent foramen ovale; TTE, transthoracic echocardiography; cTTE, contrast-enhanced transthoracic echocardiography; MIDAS, migraine disability assessment questionnaire.

A total of 168 patients with migraine who underwent PFO closure were initially screened. Four patients with grade I right-to-left shunt were excluded, leaving 164 patients eligible for further evaluation. These patients were divided into two groups based on the type of occluder: the nitinol group (*n* = 81) and the bioabsorbable group (*n* = 83). In the nitinol group, four patients were excluded due to either massive cerebral infarction within the previous 4 weeks (*n* = 3) or participation in other clinical trials (*n* = 1), resulting in 77 patients included in the final analysis. In the bioabsorbable group, two patients were excluded for recent massive cerebral infarction, leaving 81 patients in the final cohort (Figure 1).

All included patients were followed up at baseline, 1 month, 3 months, 6 months, and 12 months post-procedure. At designated follow-up visit, assessments included transthoracic echocardiography (TTE), contrast-enhanced TTE (cTTE), and the Migraine Disability Assessment Scale (MIDAS) to evaluate residual shunt and migraine severity respectively. Patient retention at each time point was recorded, with 68 patients in the nitinol group and 71 in the bioabsorbable group completing the 12-month follow-up.

The two groups were generally well-matched in terms of demographic and clinical characteristics, except for significant differences in age and serum creatinine (Table 1). The proportion of male patients was comparable between the nitinol group (37.66%) and the bioabsorbable group (25.93%; *P* = 0.113). Participants in the bioabsorbable occluder group were significantly younger than those in the nitinol group (45.58 vs. 51.55 years, *P* = 0.009). No significant differences were observed between groups in height, weight, BMI, clinical comorbidities (hypertension, diabetes, cerebral infarction, hyperlipidemia), smoking status, prior cardiac history, physiological parameters (HR, SBP, DBP) or antiplatelet usage. Liver enzyme levels (AST, ALT) were marginally higher in the nitinol group, with only AST reaching statistical significance (*P* = 0.040). Creatinine levels were slightly lower in the nitinol group (*P* = 0.003), while CRP levels showed no significant difference.

**Table 1:** Baseline characteristics.

**Clinical characteristics**	**Nitinol group (*n* = 77)**	**Bioabsorbable group (*n* = 81)**	***P* value**
Male, *N* (%)	29 (37.66%)	21 (25.93%)	0.113
Age (years)	51.55 ± 13.85	45.58 ± 13.93	0.009
Height (cm)	164.53 ± 8.13	164.11 ± 7.60	0.734
Weight (kg)	65.34 ± 13.37	62.69 ± 12.34	0.124
BMI (kg/m^2^)	24.03 ± 3.99	23.17 ± 3.54	0.145
Smoke, *N* (%)	8 (10.40%)	5 (6.20%)	0.335
Hypertension, *N* (%)	22 (28.57%)	25 (30.86%)	0.753
Diabetes, *N* (%)	5 (6.49%)	8 (9.90%)	0.439
Cerebral infarction, *N* (%)	11 (14.29%)	11 (13.58%)	0.898
Hyperlipidemia, *N* (%)	9 (11.69%)	12 (14.81%)	0.563
Antiplatelet agents, *N* (%)	77 (100.00%)	78 (96.30%)	0.262
Other cardiac surgery, *N* (%)	5 (6.50%)	3 (3.70%)	0.662
Other cardiac events, *N* (%)	16 (20.8%)	14 (17.30%)	0.576
HR (bpm)	88.55 ± 19.81	85.81 ± 13.21	0.835
SBP (mmHg)	127.17 ± 16.29	129.46 ± 18.98	0.437
DBP (mmHg)	82.04 ± 10.53	83.39 ± 11.16	0.436
BNP (pg/mL)	49.68 ± 39.12	79.20 ± 74.69	0.016
Cr (μmol/L)	65.56 ± 14.55	59.82 ± 15.78	0.003
AST (U/L)	20.70 ± 11.15	17.75 ± 8.91	0.040
ALT (U/L)	24.68 ± 18.76	19.84 ± 13.17	0.081
CRP (mg/L)	2.02 ± 1.05	2.53 ± 2.38	0.320

Values are mean ± SD or *n* (%). BMI, body mass index; HR, heart rate; SBP, systolic blood pressure; DBP, diastolic blood pressure; BNP, brain natriuretic peptide; Cr, creatinine; AST, aspartate transaminase; ALT, alanine transaminase; CRP, C-reactive protein.

The transthoracic echocardiography parameters between the nitinol group (*n* = 77) and the bioabsorbable group (*n* = 81) were compared, and the results are summarized as follows (Table 2): there was no notable distinction in the diameter of the left atrium (LA) in the nitinol group compared to the bioabsorbable group (31.49 ± 2.36 mm vs. 32.00 ± 2.60 mm, *P* = 0.225), and left ventricular systolic diameter (LVSD) and left ventricular end-diastolic diameter (LVED) were comparable between groups (*P* = 0.068 and *P* = 0.073, respectively). Both groups demonstrated normal left ventricular ejection fraction (LVEF), with no significant difference between the nitinol group (65.49% ± 1.68%) and the bioabsorbable group (66.01% ± 1.14%, *P* = 0.108). The pulmonary artery systolic pressure (PASP) was similar in both groups (22.93 ± 7.14 mmHg vs. 23.66 ± 7.36 mmHg, *P* = 0.527).

**Table 2:** Echocardiography results.

**Echocardiography results**	**Nitinol group (*n* = 77)**	**Bioabsorbable group (*n* = 81)**	***P* value**
LA (mm)	31.49 ± 2.36	32.00 ± 2.60	0.225
LVSD (mm)	30.48 ± 3.06	31.32 ± 2.53	0.068
LVED (mm)	38.40 ± 3.45	37.22 ± 3.83	0.073
LVEF (%)	65.49 ± 1.68	66.01 ± 1.14	0.108
PASP (mmHg)	22.93 ± 7.14	23.66 ± 7.36	0.527
Degree of shunt at Valsalva maneuver, *N* (%)	–	–	0.572
RLS grade II	28 (36.36%)	33 (40.74%)	–
RLS grade III	49 (63.64%)	48 (59.26%)	–

Values are mean ± SD or *n* (%). LA, left atrium; LVSD, left ventricular end-systolic diameter; LVED, left ventricular end-diastolic diameter; LVEF, left ventricular ejection fraction; PASP, pulmonary arterial systolic pressure; RLS, right-to-left shunt.

Table 3A presents the findings regarding the morphology of PFO as assessed through transesophageal echocardiography in both cohorts. The analysis revealed no statistically significant differences in parameters such as PFO separation diameter, tunnel length, the presence of atrial septal aneurysm (ASA), and the proportion of Eustachian valves when comparing the nitinol group to the bioabsorbable group (*P* > 0.05). The proportion of complex PFOs was comparable between the nitinol group and bioabsorbable group (63.64% vs. 55.56%, *P* = 0.301). The baseline PFO morphology, including diameter, tunnel length, and associated features such as atrial septal aneurysm and Eustachian valves, was well-balanced between the nitinol and bioabsorbable groups. These findings indicate that the two groups were comparable in terms of PFO complexity and structural characteristics, ensuring that subsequent outcome analyses are not influenced by baseline morphological differences.

**Table 3A:** PFO morphology.

**PFO morphology**	**Nitinol group (*n* = 77)**	**Bioabsorbable group (*n* = 81)**	***P* value**
PFO diameter (mm)	1.78 ± 0.30	1.68 ± 0.38	0.093
PFO tunnel length (mm)	8.55 ± 3.96	8.71 ± 3.78	0.756
Complex PFO, *N* (%)	49 (63.64%)	45 (55.56%)	0.301
Atrial septal aneurysm, *N* (%)	11 (14.29%)	12 (14.81%)	0.925
Eustachian valves (%)	2 (2.60%)	1 (1.23%)	0.965
Long tunnel, *N* (%)	41 (53.25%)	42 (51.85%)	0.861
Atrial septal aneurysm and Eustachian Valves (%)	1 (1.30%)	0 (0)	0.487
Atrial septal aneurysm and Long tunnel, *N* (%)	2 (2.60%)	9 (11.11%)	0.036

Values are mean ± SD or *n* (%). PFO, Patent foramen ovale. The morphology of PFOs were measured using transesophageal echocardiography.

### 3.2 Procedural information

Table 3B provides a comparative analysis of procedural data between the nitinol group and the bioabsorbable group. The selection of the occluder model was influenced by the width of the PFO separation, the length of the tunnel, and its configuration, the patient's choice of occluder material is voluntary, provided that the size and morphology are appropriate. In the Nitinol group, the most frequently used occluder model and size was 24/24 mm, accounting for 62.34% of cases. The second most commonly used device was the AMPLATZER™ occluder 18/25 mm, which features asymmetric left and right disks. The most common occluder model in the bioabsorbable group was also 24/24 mm (83.95%), due to the absence of 18/25 mm devices in this group. The smallest occluder (18/18 mm) was used in a comparable proportion of cases in both groups (9.09% in the nitinol group vs. 8.64% in the bioabsorbable group). The 18/18 mm and 34/34 mm occluders were the least frequently utilized device sizes in both the nitinol and bioabsorbable groups, reflecting their limited application in the studied population.

**Table 3B:** Procedural data.

**Procedural data**	**Nitinol group (*n* = 77)**	**Bioabsorbable group (*n* =81)**	***P* value**
**Occluder size**, ***N*** **(%)**			
18/18 mm	7 (9.09%)	7 (8.64%)	<0.001
24/24 mm	48 (62.34%)	68 (83.95%)	
18/25 mm	18 (23.38%)	0 (0)	
28/28 mm	2 (2.60%)	6 (7.41%)	
34/34 mm	2 (2.60%)	0 (0)	
Procedure time (min)	31.61 ± 2.89	32.51 ± 3.43	0.095
Fluoroscopy time (s)	284.35 ± 96.78	311.41 ± 103.01	0.091
Radiation dosage (mGy)	35.04 ± 13.68	38.91 ± 20.35	0.547
Inpatient days (day)	4.54 ± 2.36	4.56 ± 2.47	0.169
Success rate, *N* (%)	100	100	–

Values are mean ± SD or *n* (%).

No statistically significant variation was observed in the duration of X-ray exposure during the procedure or radiation dosage between the nitinol group and the bioabsorbable group (fluoroscopy times 284.35 ± 96.78 sec and 311.41 ± 103.01 sec, respectively, *P* = 0.091; and radiation dosage 35.04 ± 13.68 mGy and 38.91 ± 20.35 mGy, respectively, *P* = 0.547). The procedural time was slightly shorter in the nitinol group (31.61 ± 2.89 min) compared to the bioabsorbable group (32.51 ± 3.43 min), though this difference was not statistically significant (*P* = 0.095). The average inpatient stay was similar between groups, with 4.54 ± 2.36 days in the nitinol group and 4.56 ± 2.47 days in the bioabsorbable group (*P* = 0.169). Both groups achieved a 100% procedural success rate, indicating the effectiveness of both occluder types in PFO closure. The procedural characteristics between the nitinol and bioabsorbable groups were largely comparable, with both achieving excellent procedural success rates. Despite slight differences in procedural time, this variation was not statistically significant, underscoring the overall feasibility of both occluder types in clinical practice.

### 3.3 Safety and complications

Both nitinol and bioabsorbable occluders demonstrated excellent safety profiles with low rates of perioperative complications (Table 4). In both the nitinol group and the bioabsorbable group, there were no occurrences of sudden cardiac arrest, cardiac tamponade, endocarditis, or thrombus formation on the device reported during or within 24 h following the occlusion. The only statistically significant difference was a single case of occluder detachment in the bioabsorbable group. The rates of vascular complications and new arrhythmias were comparable between the groups, and no neurological events occurred.

Postoperative arrhythmias were infrequent complications associated with the devices in both cohorts. New arrhythmias after implantation were observed in 3 patients (3.90%) in the nitinol group and 5 patients (6.17%) in the bioabsorbable group, with no statistically significant difference (*P* = 0.772). The specific types of arrhythmias observed included transient atrial fibrillation (AF), which occurred in 2.60% of cases in the nitinol group and 3.70% in the bioabsorbable group, and premature contractions, reported in 1.30% of the nitinol group and 3.70% of the bioabsorbable group. All arrhythmia events resolved with conservative treatment. No instance of complete atrioventricular block occurred in either cohort throughout the 12-month follow-up duration. These findings highlight the overall safety of both occluder types for PFO closure.

**Table 4:** Perioperative results.

**SADEs following implantation**	**Nitinol group (*n* =77)**	**Bioabsorbable group (*n* = 81)**	***P* value**
Sudden cardiac arrest, *N* (%)	0 (0)	0 (0)	–
Cardiac tamponade, *N* (%)	0 (0)	0 (0)	–
Occluder falling off, *N* (%)	0 (0)	1 (1.23%)	1.000
Endocarditis, *N* (%)	0 (0)	0 (0)	–
Thrombus formation on the device, *N* (%)	0 (0)	0 (0)	–
Vascular complication	3 (4.00%)	2 (2.47%)	0.954
New arrhythmia after implantation, *N* (%)	3 (3.90%)	5 (6.17%)	0.772
AVB, *N* (%)	0 (0)	0 (0)	–
AF, *N* (%)	2 (2.60%)	3 (3.70%)	1.000
Premature contractions (%)	1 (1.30%)	3 (3.70%)	0.649
**Neuro events after PFO closure**			
TIA, *N* (%)	0 (0)	0 (0)	–
Stroke, *N* (%)	0 (0)	0 (0)	–

Values are mean ± SD or *n* (%). SADEs, serious adverse device effects; AVB, atrioventricular block; AF, atrial fibrillation; TIA, transient ischemic attack.

### 3.4 Degradation of the bioabsorbable occluder

**Figure 2:**
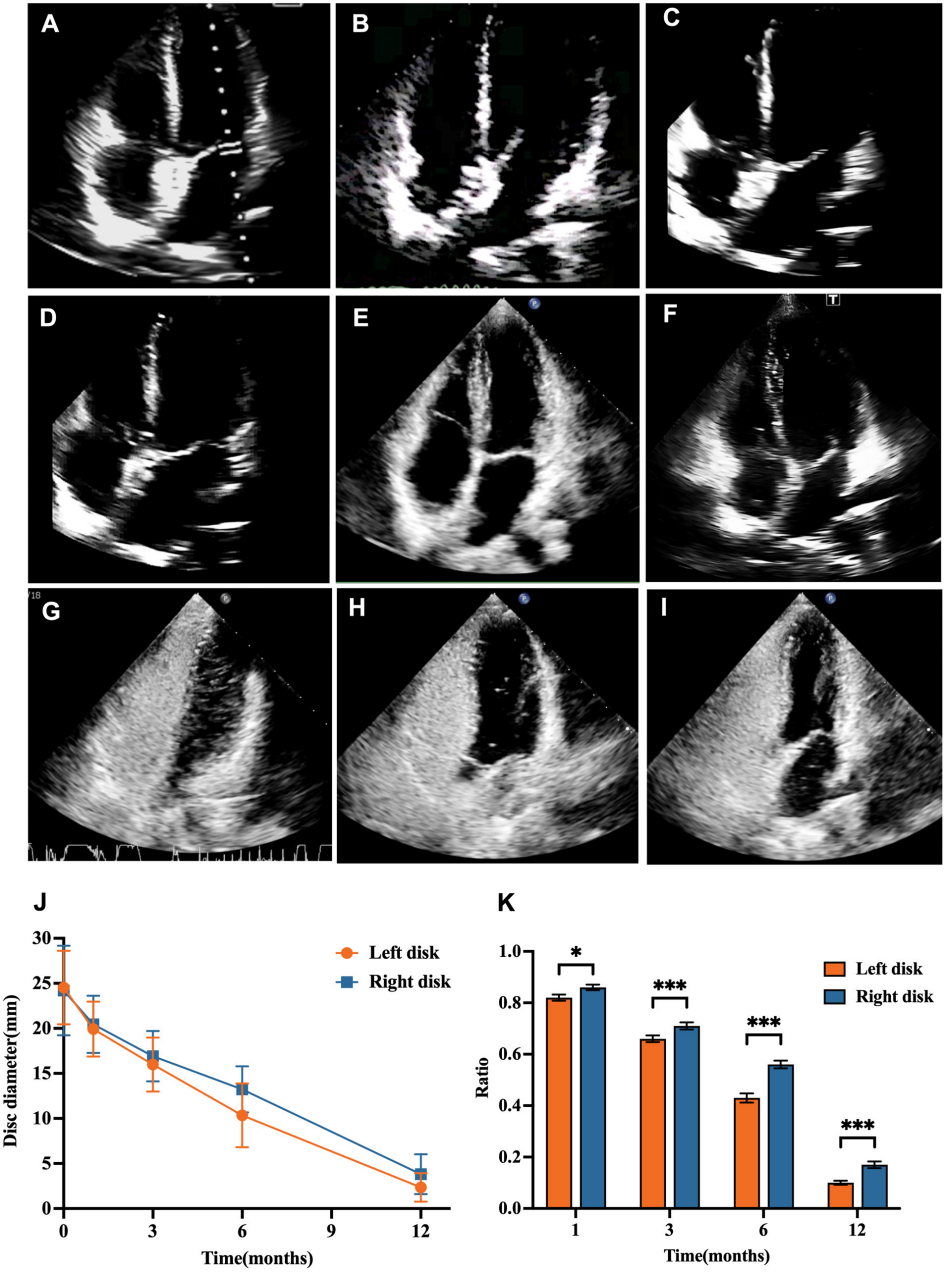
Morphological alterations of the entirely bioabsorbable occluder, as evidenced by TTE over the course of a 12-month follow-up period. **(A–F)** The TTE four-chamber view was assessed post-discharge, as well as at the 1-, 3-, 6-, 9-, and 12-month follow-ups, respectively. **(G–I)** cTTE images were captured prior to closure and at the 6- and 12-month follow-ups, respectively. **(J)** A quantitative evaluation of the diameter of the fully bioabsorbable occluder as illustrated by TTE throughout the 12-month follow-up. **(K)** The ratio of the disk area measured at the 1-, 3-, 6-, and 12-month follow-ups compared to that measured before discharge in the five-chamber view. ^*^P < 0.05; ^***^P < 0.001. The error bar represents the standard error of the mean.

The degradation process of bioabsorbable occluders was evaluated through echocardiographic imaging. Left and right disk values measured at each follow-up visit. The data demonstrate that biodegradable occluders progressively reduce in size due to their bioabsorbable nature. This degradation process highlights the distinct characteristics of the two types of occluders post-implantation. The bioabsorbable occluder was observed on echocardiography as a hyperechoic area protruding from the adjacent endocardium at 3 months; however, by the 6-month follow-up, it was level with the surrounding endocardium (Figures 2A–F). Notably, at the 12-month follow-up, echocardiography revealed no hyperechoic region on the atrial septum in certain patients. In the TTE four-chamber view, the diameter of the left disk diminished from 24.60 ± 4.09 mm at discharge to 2.35 ± 1.58 mm at 12 months, while the right disk experienced a reduction from 24.30 ± 5.08 mm to 3.82 ± 2.21 mm, indicating a more rapid degradation of the left disk compared to the right (Figures 2J, K). The degradation process was quantified through the reduction in the area of the hyperechoic region, which was evident in both disks of the biodegradable occluder. The reduction in size of the biodegradable occluders is a key feature, reflecting the gradual resorption of the material, which may be beneficial for long-term outcomes in certain patient populations. See Supplementary Table S1 for details.

### 3.5 Residual shunt after closure

**Figure 3:**
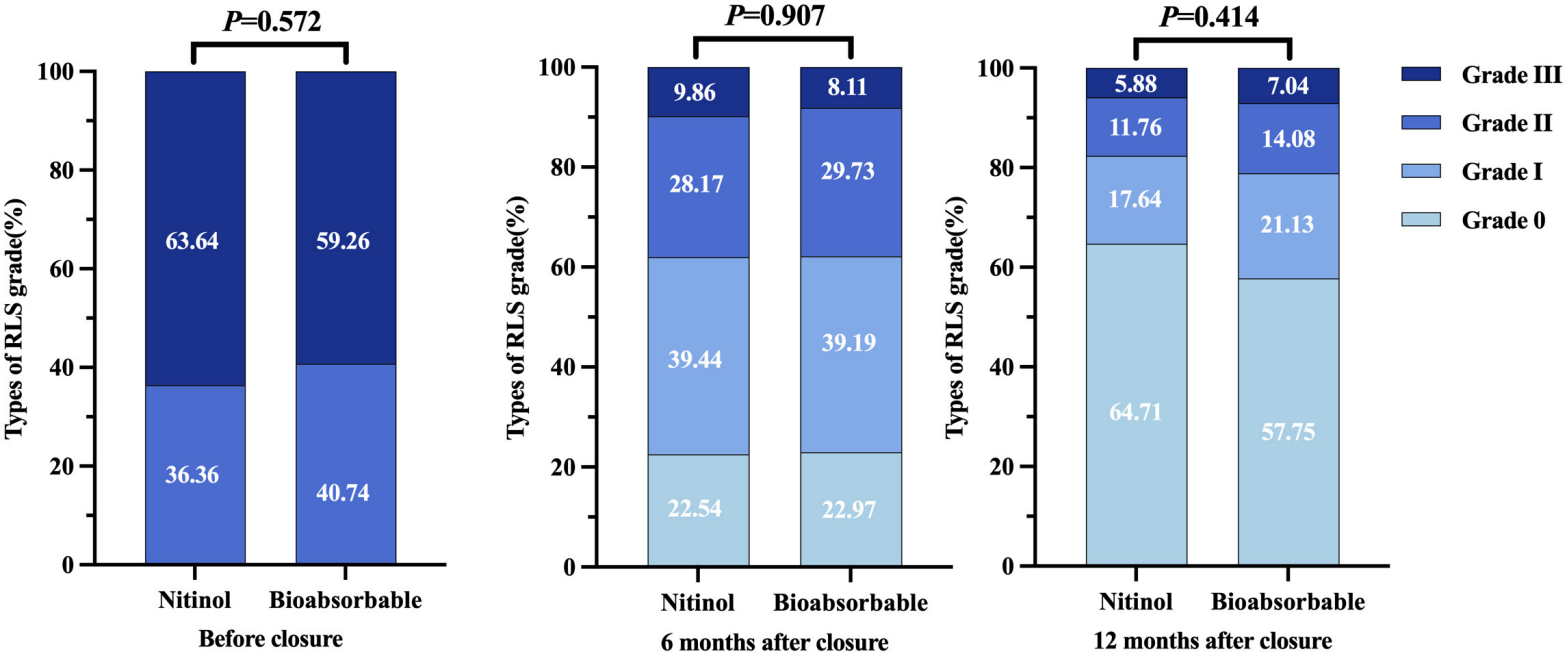
The RLS grading classified by cTTE. The RLS change were detected before closure, and at 6-, 12-month follow-up. RLS, right-to-left shunt.

The analysis in Supplementary Table S2 compares the residual RLS grades between the nitinol and bioabsorbable groups before and after percutaneous PFO closure at 6 and 12 months. The majority of patients in both groups presented with RLS grade III before closure, with 63.64% in the nitinol group and 59.26% in the bioabsorbable group. RLS grade II was observed in 36.36% of the nitinol group and 40.74% of the bioabsorbable group (Figure 3). There were no significant differences in the distribution of RLS grades between the groups before closure (*P* = 0.572), at 6 months post-closure (*P* = 0.907), or at 12 months (*P* = 0.414). At 6 months, the proportion of patients with no residual shunt (RLS grade 0) increased to 22.54% in the nitinol group and 22.97% in the bioabsorbable group. The proportion of patients with RLS grade I increased to 39.44% in the nitinol group and 39.19% in the bioabsorbable group. RLS grades II and III decreased significantly in both groups, with 9.86% in the nitinol group and 8.11% of patients in the bioabsorbable group respectively, having RLS grade III. 28.17% in the nitinol group and 29.73% of patients in the bioabsorbable group respectively, having RLS grade II (Figure 3). At 12 months, the majority of patients achieved complete closure (RLS grade 0), with rates of 64.71% in the nitinol group and 57.75% in the bioabsorbable group. Only a small fraction of patients remained with residual shunting: 11.76% with RLS grade II and 5.88% with RLS grade III in the nitinol group, and similar proportions (14.08% with RLS grade II and 7.04% with RLS grade III) in the bioabsorbable group (Figure 3). Both groups achieved high rates of complete closure (RLS grade 0), with a slightly difference not statistically significant, indicating that both occluder types were effective in achieving PFO closure. The image change in RLS after closure demonstrated by cTTE is illustrated in Figures 2G–I.

### 3.6 Improvement in migraine after PFO closure

**Figure 4:**
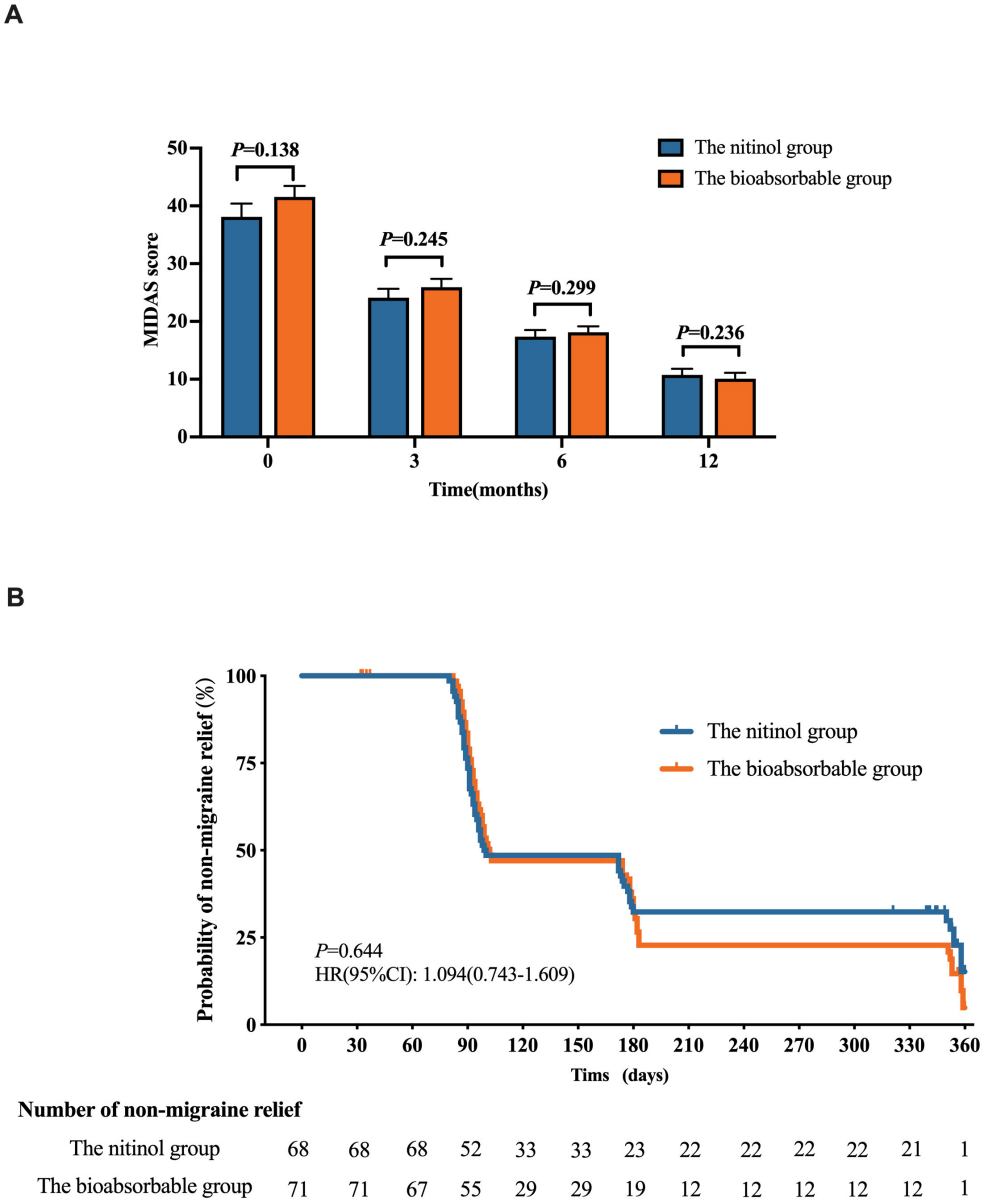
**(A)** MIDAS score measured before procedure, 3-, 6-, and 12-month follow-up assessments following occlusion. **(B)** The Kaplan-Meier (KM) curve compares the rates of non-migraine relief between the nitinol group and the bioabsorbable group.

To clarify the therapeutic efficacy of occluder implantation in patients with PFO-related migraine, follow-up evaluations were conducted. Post-procedural assessments revealed no increase in either the dosage or frequency of migraine-specific medication use compared to pre-procedural levels. Prior to occlusion, no notable differences were observed in the MIDAS scores when comparing the nitinol group to the biodegradable group (*P* = 0.138; Supplementary Table S3). Both groups experienced significant improvements in migraine severity after PFO closure. In the biodegradable group, MIDAS scores decreased from 41.54 ± 17.18 before closure to 10.07 ± 8.81 at 12 months after closure. While in the nitinol group, MIDAS scores decreased from 38.09 ± 20.26 to 10.74 ± 8.81 at 12 months after closure (*P* < 0.01). During the follow-up period, no statistically significant differences were detected between the two groups regarding post-procedure MIDAS scores at the 3-, 6- and 12-month follow-up assessments following occlusion (*P* = 0.245; *P* = 0.299; *P* = 0.236, respectively; Figure 4A).

The Kaplan-Meier (KM) curve compares the rates of non-migraine relief between the nitinol group and the bioabsorbable group (Figure 4B). The analysis is based on a criterion for migraine relief, defined as a ≥50% reduction in MIDAS score. The comparison between the two groups indicates no statistically significant difference in migraine relief rates between the two groups over the 12-month follow-up period (*P* = 0.644). At the 12-month mark, both groups had achieved significant relief in the majority of patients, this suggests that both occluder types were equally effective in providing migraine relief in the study population.

Moreover, we performed a subgroup analysis to assess this association. Among patients with moderate-to-severe residual shunt after PFO closure, 58.3% reported no improvement in migraine symptoms. This proportion is notably higher compared to the 41.7% non-responders observed in patients with either no residual shunt or only mild residual shunt. The difference between these groups was statistically significant (*P* < 0.001). These findings suggest that the presence of a significant residual shunt may be associated with a reduced therapeutic effect of PFO closure on migraine, thus supporting the importance of complete or near-complete closure in achieving optimal clinical outcomes.

## 4 Discussion

The major findings of the retrospective cohort study suggest that both nitinol and degradable PFO occluders are equally effective in alleviating migraine severity in selected PFO patients, as shown by improvements in MIDAS scores over a 1-year follow-up. Both occluder types demonstrated excellent safety profiles, with low rates of perioperative complications. Consequently, in the management of PFO patients with migraine, the novel biodegradable occluder appears to offer therapeutic advantages comparable to those of traditional nitinol occluders.

The fundamental traits of the study cohort were observed to be comparable between the nitinol and biodegradable occluder groups. The only significant differences between the two groups observed were related to age and serum creatinine levels. Specifically, the biodegradable occluder group was found to be significantly younger and exhibited lower serum creatinine levels. Because of concerns about the long-term presence of permanent foreign materials, which can result in chronic inflammation or device-related complications, younger patients may prefer biodegradable occluders. Younger patients, often more active and health-conscious, may favor devices that minimize these risks, and biodegradable occluders provide the benefit of gradual resorption over time. Notably, this observed discrepancy in age did not result in substantial disparities across other baseline clinical or physiological parameters. These findings support the conclusion that the two groups were comparable at baseline, and any differences in outcomes observed can be attributed to the type of occluder used rather than underlying demographic or clinical disparities. Moreover, this study suggest that among patients with migraine associated with PFO, females are disproportionately represented, indicating a higher prevalence in women (29).

Procedural outcomes, including occluder model and size selection, procedural time, and hospital stay duration, were largely comparable between the two groups. The procedural success rate was 100% in both the biodegradable and nitinol occluder groups, demonstrating the feasibility and safety of both devices in PFO closure. The slight reduction in procedural time observed in the biodegradable group was not statistically significant, suggesting that the biodegradable occluder can be used in clinical practice without additional procedural burden. Radiation exposure times and doses were also similar between the groups, further supporting the overall procedural comparability of both devices.

Currently, in terms of occluder-related thrombus formation, biodegradable occluders have only recently been introduced into clinical practice, and thus, long-term safety data remain limited. To ensure patient safety, our antiplatelet therapy regimen (DAPT type and duration) was aligned with that used for metallic occluder devices in this study. We agree that one potential advantage of biodegradable devices could be the possibility of shortening DAPT duration. However, we believe that any modification to antiplatelet strategies should be based on robust long-term follow-up data. We will update the manuscript to include a discussion of this consideration and the need for future studies to evaluate optimal DAPT regimens in patients receiving biodegradable devices. In our study, follow-up transesophageal echocardiography (TEE) was not routinely performed; instead, transthoracic echocardiography (TTE) and contrast transthoracic echocardiography (cTTE) were used during follow-up evaluations. While TTE is capable of detecting obvious thrombi, it may not identify smaller or more subtle thrombi that TEE could potentially reveal. This methodological difference may partially explain why our reported postoperative thrombus formation rate is lower compared to the rates reported in other studies, such as those by Du et al. (23) and He et al. (25).

The only notable complication was a single case of occluder detachment in the biodegradable group, which occurred in a single patient and did not significantly impact the overall safety profile of the biodegradable occluder. The patient had a long-tunnel type PFO with a tunnel length of 11 mm and no other anatomical abnormalities. The failure was was not related to the PFO anatomical features or the choice of occluder. The case of occluder detachment was likely due to the failure to promptly remove the connecting line during the procedure, which can hinder proper device deployment and stabilization. During the implantation process, if the shape line is not adequately retracted after positioning the occluder, it may create mechanical stress or displace the device, potentially leading to detachment. Fortunately, this issue was recognized promptly during the procedure, and the occluder was successfully repositioned and reimplanted a new biodegradable occluder without any significant delay or harm to the patient. The reimplantation was executed smoothly after the detachment was detected, and the patient experienced no major complications. This isolated event serves as a valuable learning point for improving procedural outcomes. A meticulous and standardized workflow for PFO occluder implantation, incorporating rigorous monitoring of connection line and occluder stability prior to removal, is paramount to averting device detachment. By acquiring familiarity with the implantation procedure and meticulously overseeing equipment and connecting wires during the implantation process, complications of a similar natuFre can be effectively circumvented. Furthermore, the successful reimplantation of biodegradable PFO occluders in this case substantiates the safety and efficacy of the device when utilized in accordance with established protocols, thereby imparting invaluable insights that can inform the enhancement of clinical practices and the mitigation of the risk of future occurrences of a similar nature.

The incidence of postoperative arrhythmias, including transient atrial fibrillation (AF) and premature contractions, was observed in both groups, and no occurrences of complete atrioventricular block were documented in either group. The arrhythmias manifested as transient episodes and resolved with conservative treatment, a finding that aligns with prior studies examining the outcomes of PFO closure (6, 30). These findings provide further evidence to support the safety of both occluder types, with no major complications related to the devices reported (31). Moreover, biodegradable occluders are theoretically anticipated to significantly lower the incidence of 'device syndrome' associated with nickel hypersensitivity after PFO closure (32). We suppose that in patients with a known history of metal allergy or suspected nickel sensitivity, careful consideration should be given before selecting a nitinol-containing device. Emerging alternatives such as biodegradable occluders may reduce the risk of nickel-related adverse effects and could represent a promising direction for future device development (33). However, this study did not assess such outcomes due to the absence of well-defined diagnostic criteria for nickel-induced device syndrome. While large-scale evidence is still lacking, awareness of nickel hypersensitivity is important in the pre-procedural evaluation and long-term management of patients undergoing PFO closure.

Both the nitinol and biodegradable occluders achieved high rates of complete PFO closure, with no significant differences between the two groups in terms of residual shunting at 6 and 12 months. This demonstrates the efficacy of both occluder types in achieving PFO closure. The reduction in size did not compromise the occluder's ability to maintain PFO closure, as evidenced by the high rates of RLS grade 0 and I in the biodegradable group were comparable with the nitinol group. These findings align with previous studies on biodegradable occluders and support the clinical efficacy of both device types in achieving effective PFO closure, highlighting that both provide durable and reliable outcomes.

The two occluder types exhibited a marked reduction in migraine severity at 6 and 12 months post-procedure. The biodegradable occluder group exhibited a decrease in MIDAS score that was comparable to the nitinol group. thereby suggesting the efficacy of biodegradable devices in the treatment of migraines associated with PFO. Importantly, the absence of statistically significant differences in the improvement of migraine between the two groups suggests that biodegradable devices offer a comparable therapeutic effect to nitinol occluders. Moreover, our reported residual shunt rate is higher than those reported by Du et al. (23) (4.5% for severe residual shunt), He et al. (25) (3% for moderate to substantial residual shunt). This discrepancy may be explained by several factors. Given the high prevalence of complex anatomical features in our study, anatomical variations are known to be associated with increased technical difficulty during closure and a higher likelihood of residual shunt. In another study (Chunying Ji et al. Chinese Circulation Journal 2024), our data were relatively consistent with their findings (34). Given that reasonable differences may exist between centers, further exploration of post-procedural RLS will benefit from multicenter research data. In addition, study from Ilkay Erdogan et al. have showed that closing a PFO via septostomy reduced the residual-shunt rate in long-channel-type PFOs (35). Including this information could suggest that, in future studies, using a septostomy approach rather than crossing the channel may make biodegradable PFO devices safer.

Although our findings suggest that the presence of a significant residual shunt may be associated with a reduced therapeutic effect of PFO closure in patients with migraine, highlighting the importance of achieving complete or near-complete closure for optimal clinical outcomes. However, we also observed that in some cases, migraine symptoms did not improve despite a substantial reduction in residual shunt. This indicates that migraine relief cannot be explained solely by the extent of residual shunt. Further studies with larger sample sizes, detailed univariate and multivariate regression analyses, as well as subgroup analyses, are needed to identify additional factors that may influence the prognosis of migraine following PFO closure, such as device size and migraine subtype (with or without aura).

This study highlights the growing relevance of biodegradable occluders as a viable alternative to nitinol devices for PFO closure, particularly in younger patients or those with concerns about long-term foreign material retention. The degradation process of the biodegradable occluder was evaluated through echocardiography, revealing a consistent and anticipated gradual decrease in the device's size over time. This gradual degradation process coincided with endothelialization, leaving behind native tissue without residual structural abnormalities, which is in line with the device's design and intended function to minimize the long-term presence of foreign material, which may benefit long-term outcomes, particularly for younger patients or those with concerns about lifelong implants. In our study, we hypothesized several potential mechanisms that could contribute to the differential degradation rates observed between the left and right disks of the biodegradable occluder. One possibility is that variations in the local microenvironment, such as differences in pH or enzymatic activity, could affect the degradation rate. Additionally, the left disk may have a different exposure to blood flow dynamics or shear stress, which could influence its degradation. Meanwhile, the comparable efficacy and safety profiles of the two devices allow clinicians to tailor device selection to individual patient preferences and clinical scenarios. As biodegradable technology continues to evolve, it holds promise for expanding the therapeutic options available for patients undergoing PFO closure.

Nevertheless, this study is subject to several limitations. Firstly, its retrospective design and single-center setting may limit the generalizability of the findings. Furthermore, the relatively brief follow-up period may not encompass long-term outcomes or complications. Consequently, prospective, multicenter studies with extended follow-up are necessary to validate these findings and further explore the long-term benefits of biodegradable occluders in migraine management.

## 5 Conclusion

Both nitinol occluders and biodegradable are effective and safe for selected PFO closure in migraine treatment, with comparable outcomes in procedural success, safety, and migraine alleviation. The biodegradable occluder offers the added advantage of resorption, reducing the risks associated with long-term device retention. These findings provide robust evidence supporting the use of biodegradable occluders as a promising alternative to nitinol devices. Further research is warranted to confirm these results and refine patient selection criteria to optimize outcomes.

## Data Availability

The original contributions presented in the study are included in the article/Supplementary material, further inquiries can be directed to the corresponding authors.
