# Supplementary material for: Comparative efficacy and safety of nitinol vs. novel fully biodegradable occluders for transcatheter patent foramen ovale closure in migraine treatment: a retrospective cohort study
Source: Front Med (Lausanne). 2025 Jul 2;12:1613687. doi: 10.3389/fmed.2025.1613687 (PMC12263644; doi:10.3389/fmed.2025.1613687)
Supplement: Supplementary file 1 [file Table_1.docx]

Supplementary Material

# Supplementary tables

**Table S1 Size and thickness change of bioabsorbable occluder plate after closure.**

|  | n | Left disk (mm) | Right disk (mm) | *P* value |
| --- | --- | --- | --- | --- |
| Postoperative day 1 | 81 | 24.60±4.09 | 24.30±5.08 | 0.646 |
| 1 months after closure | 81 | 20.05±2.91 | 20.41±3.19 | 0.280 |
| 3 months after closure | 76 | 16.06±2.83 | 16.87±2.81 | 0.049 |
| 6 months after closure | 74 | 10.35±3.52 | 13.19±2.60 | <0.001 |
| 12 months after closure | 71 | 2.35±1.58 | 3.82±2.21 | <0.001 |

Values are mean ± SD or n (%).

**Table S2 Change of RLS after closure.**

|  | Nitinol group  (n =77) | Bioabsorbable group  (n =81) | *P* value |
| --- | --- | --- | --- |
| RLS before closure, N (%) |  |  | 0.572 |
| RLS grade 0 | 0 | 0 | - |
| RLS grade I | 0 | 0 | - |
| RLS grade II | 28(36.36%) | 33(40.74%) | - |
| RLS grade III | 49(63.64%) | 48(59.26%) | - |
|  | Nitinol group  (n =71) | Bioabsorbable group  (n =74) | *P* value |
| Residual shunt  6 months after closure, N (%) |  |  | 0.907 |
| RLS grade 0 | 16(22.54%) | 17(22.97%) | - |
| RLS grade I | 28(39.44%) | 29(39.19%) | - |
| RLS grade II | 20(28.17%) | 22(29.73%) | - |
| RLS grade III | 7(9.86%) | 6(8.11%) | - |
|  | Nitinol group  (n =68) | Bioabsorbable group  (n =71) | *P* value |
| Residual shunt  12 months after closure, N (%) |  |  | 0.414 |
| RLS grade 0 | 44(64.71%) | 41(57.75%) | - |
| RLS grade I | 12(14.71%) | 15(21.13%) | - |
| RLS grade II | 8(11.76%) | 10(14.08%) | - |
| RLS grade III | 4(5.88%) | 5(7.04%) | - |

Values are mean ± SD or n (%).

**Table S3 Improvement in migraine symptom after PFO closure.**

|  | Nitinol group  (n =77) | Bioabsorbable group  (n =81) | *P* value |
| --- | --- | --- | --- |
| MIDAS score before closure | 38.09±20.26 | 41.54±17.18 | 0.138 |
|  | Nitinol group  (n =72) | Bioabsorbable group  (n =76) | *P* value |
| MIDAS score 3 months after closure | 24.12±12.94 | 25.92±12.56 | 0.245 |
|  | Nitinol group  (n =71) | Bioabsorbable group  (n =74) | *P* value |
| MIDAS score 6 months after closure | 17.37±9.72 | 18.11±8.89 | 0.299 |
|  | Nitinol group  (n =68) | Bioabsorbable group  (n =71) | *P* value |
| MIDAS score 12 months after closure | 10.74±8.81 | 10.07±8.81 | 0.236 |

 Values are mean ± SD or n (%). MIDAS, Migraine Disability Assessment Questionnaire.

# Supplementary videos

**Video 1.** Intracardiac echocardiography (ICE)-guided real-time imaging of guidewire passage through PFO.

**Video 2.** The left disc of the occluder is initially released in a spherical shape from the delivery sheath into the left atrium.

**Video 3.** The right disc is released after confirming the proper configuration of the left disc.

**Video 4.** The entire occluder takes on a "double-umbrella" shape.
